# A systematic review on multifocal non-invasive brain stimulation on motor function enhancement

**DOI:** 10.3389/fnhum.2026.1779424

**Published:** 2026-04-20

**Authors:** Won Kee Chang, Ji Soo Choi, Won-Seok Kim, Nam-Jong Paik

**Affiliations:** Department of Rehabilitation Medicine, Seoul National University College of Medicine, Seoul National University Bundang Hospital, Seongnam-si, Republic of Korea

**Keywords:** brain stimulation, motor, neuromodulation, transcranial current stimulation, transcranial electric stimulation, transcranial magnetic stimulation

## Abstract

**Background:**

Multifocal non-invasive brain stimulation (NIBS) has been highlighted as a promising approach for modulating motor functions by simultaneously stimulating several brain areas. However, the effectiveness and underlying mechanisms of this technique are still not well understood.

**Objective:**

To evaluate the effectiveness of multifocal NIBS in promoting motor recovery, considering the targeted brain regions, stimulation protocols, and both behavioral and neurophysiological outcomes.

**Methods:**

We conducted a systematic search of the PubMed, EMBASE, Cochrane Library, Scopus, and Web of Science databases up to February 10, 2025, adhering to the Preferred Reporting Items for Systematic Reviews and Meta-Analyses 2020 guidelines. The inclusion criteria encompassed human studies utilizing concurrent dual-site or multifocal NIBS with pre- and post-intervention behavioral assessments. Studies on deep brain stimulation, corticocortical paired associative stimulation, sequential stimulation, case reports, and reviews were excluded.

**Results:**

Out of 1,453 initial records, 9 studies met the inclusion criteria. These studies primarily focused on stimulating the dorsolateral prefrontal cortex and primary motor cortex. Neurophysiological data obtained from electroencephalography and functional magnetic resonance imaging indicated network-level modulations. Behavioral outcomes were variable, with some studies showing improvements in response inhibition, and motor learning, while others found no significant differences compared to sham stimulation.

**Conclusion:**

While multifocal NIBS shows promise as a novel therapeutic approach, its efficacy remains uncertain due to variations in study designs, small sample sizes, and inconsistent results. Future research should focus on larger sample size, multi-arm trials and inclusion of neurophysiological biomarkers.

**Systematic review registration:**

https://www.crd.york.ac.uk/PROSPERO/view/CRD420250646196, identifier: CRD420250646196.

## 1 Introduction

Non-invasive brain stimulation (NIBS), which includes techniques such as repetitive transcranial magnetic stimulation (rTMS), transcranial electrical stimulation (tES)-encompassing transcranial direct current stimulation (tDCS), and transcranial alternating current stimulation (tACS)-has emerged as an innovative tool for promoting functional recovery in individuals with neurological disorders (Schulz et al., 2013). This field has shown rapid advances owing to the development of new technologies and stimulation protocols. Over the past few decades, NIBS has shown promise for treating neurological conditions, including stroke (Xiang et al., 2019; Li et al., 2024; Falaschi et al., 2025) and neurodegenerative diseases (Lee et al., 2024; Harris et al., 2025). However, the clinical outcomes remain inconsistent, with ongoing challenges in establishing robust effects and determinating the optimal candidates for treatment (Sanches et al., 2020).

Complex motor functions are now increasingly understood to depend on the simultaneous activation and synchronization of multiple brain networks, rather than being confined to a single brain region (Sale et al., 2015). To address this, multifocal NIBS has been introduced as an innovative approach that targets these functional networks rather than isolated regions (Fischer et al., 2017). For the purposes of this review, multifocal NIBS refers to protocols that deliver stimulation to two or more anatomically distinct brain regions concurrently (i.e., simultaneously or with overlapping stimulation windows), targeting functionally or structurally connected nodes of a neural network. Unlike traditional monofocal NIBS, which primarily modulates activity in a single brain area, multifocal NIBS focuses on enhancing interactions across multiple brain networks, potentially leading to more consistent and robust functional improvements (Menardi et al., 2022).

Although there is increasing interest in multifocal NIBS (Wessel et al., 2021, 2024; Rektorová et al., 2025), systematic reviews on its clinical outcomes are lacking. Therefore, this review seeks to consolidate the existing literature on multifocal NIBS related to motor functions, focusing specifically on the targeted brain regions, the stimulation techniques used (such as stimulation parameters, electrode configurations, and coil placements), and the resulting behavioral outcomes.

This study aimed to assess the current evidence level by systematically reviewing the existing literature and to suggest future research directions in applying of multifocal NIBS to improve motor functions.

## 2 Methods

### 2.1 Eligibility criteria

This systematic review was conducted following the latest version of the Preferred Reporting Items for Systematic Reviews and Meta-Analyses (PRISMA) 2020 guidelines (Page et al., 2021). The review comprised peer-reviewed research articles and preprints published in English that examined the effects of concurrent dual-site or multifocal non-invasive brain stimulation (NIBS) in human participants. Eligible studies were required to report behavioral outcomes related to motor functions assessed before and after the intervention. Excluded from the review were simulation studies, case reports, retrospective studies, review articles, and non-research publications such as conference abstracts. Furthermore, studies investigating stimulation techniques other than NIBS, including deep brain stimulation (DBS) were excluded. Paired associative stimulation (PAS), or corticocortical PAS (ccPAS), were also not considered. We excluded studies that used multi-electrode high-definition stimulation targeting a single brain region, as this falls outside the scope of our review. Studies utilizing cortico-cortical Paired Associative Stimulation (ccPAS) were excluded from this review. While ccPAS aims to induce Hebbian-type spike-timing-dependent plasticity (STDP), it relies on the sequential activation of two nodes via a fixed interstimulus interval. This stands in methodological contrast to the concurrent, multifocal non-invasive brain stimulation (NIBS) protocols investigated here, which prioritize simultaneous modulation. Furthermore, the clinical and physiological effects of ccPAS have been extensively documented in recent literature (Hernandez-Pavon et al., 2023; Di Luzio et al., 2024). For similar reasons, we excluded sequential paradigms involving priming of same region, as well as bifocal stimulation of bilateral homologous areas, to maintain a strict focus on the simultaneous activation of distinct functional networks"

Included studies were categorized according to the type of intervention, study population and the nature of the reported outcomes.

### 2.2 Information sources

A comprehensive literature search was performed on five major electronic databases: PubMed, EMBASE, Cochrane Library, Scopus, and Web of Science. Searches were restricted to these databases and did not include general search engines or non-indexed websites. No limitations were applied regarding publication dates. The final search was completed on February 10, 2025

### 2.3 Search strategy

The search strategy was developed to identify studies investigating dual-site or multifocal non-invasive brain stimulation (NIBS). No restrictions were applied based on language, publication type, or population during the initial search. The following search terms and Boolean operators were employed in PubMed: #1 (“non-invasive”[Title/Abstract] OR “noninvasive”[Title/Abstract]) AND “stimulation”[Title/Abstract]; #2 (“transcranial”[Title/Abstract] OR “trans-cranial”[Title/Abstract]) AND “stimulation”[Title/Abstract]; #3 (“tDCS”[Title/Abstract] OR “tACS”[Title/Abstract] OR “TMS”[Title/Abstract] OR “rTMS”[Title/Abstract] OR “transcranial direct current stimulation”[MeSH Terms] OR “transcranial magnetic stimulation”[MeSH Terms]); #4 (“theta burst”[Title/Abstract] OR “theta-burst”[Title/Abstract] OR “TBS”[Title/Abstract] OR “cTBS”[Title/Abstract] OR “iTBS”[Title/Abstract]); #5 (“temporal interference stimulation”[Title/Abstract] OR “TIS”[Title/Abstract] OR “tTIS”[Title/Abstract]); #6 (“focused ultrasound”[Title/Abstract] OR “focused-ultrasound”[Title/Abstract] OR “LIFU”[Title/Abstract]); #7 (“dual-site”[Title/Abstract] OR “dual site”[Title/Abstract] OR “multifocal”[Title/Abstract] OR “multi-focal”[Title/Abstract]); (#1 OR #2 OR #3 OR #4 OR #5 OR #6) AND #7. The strategy was tailored to conform to syntax and indexing rules specific to each database to maximize relevant study retrieval.

### 2.4 Selection process

Study selection was performed independently by two reviewers, who conducted screening at each stage of the process. Initially, titles and abstracts were examined to eliminate studies not relevant to the topic. Subsequently, the eligibility of full-text articles was assessed according to predetermined inclusion and exclusion criteria. Any disagreements between reviewers were addressed through discussion until consensus was reached. No automated screening tools or software were utilized during study selection.

### 2.5 Data collection process

Data extraction was carried out independently by two reviewers utilizing a standardized data extraction form. Any discrepancies encountered during the extraction process were resolved through discussion until consensus was achieved. No automated tools or software were employed during data collection

### 2.6 Data items

The data extracted from the included studies were systematically organized into structured tables and classified into distinct categories such as publication year, authorship, and study design; intervention parameters including stimulation type, electrode configuration, intensity, frequency, and duration; participant demographics covering sample size, age, sex, and health status; and targeted functions of motor tasks. Outcome measures were further categorized into behavioral outcomes and neurophysiological results, including functional magnetic resonance imaging (fMRI), electroencephalography (EEG), and evoked potential data. The timing of assessments and details of intervention sessions were meticulously recorded. Effect sizes (Cohen's d or partial eta-squared [η^2^]) were extracted or calculated from reported statistics where feasible to supplement significance testing.

### 2.7 Risk of bias assessment

The methodological quality of the included randomized controlled trials was appraised using the Cochrane Risk of Bias tool (Higgins et al., 2011). Seven domains were evaluated in each study: (1) random sequence generation, (2) allocation concealment, (3) blinding of participants and personnel, (4) blinding of outcome assessment, (5) incomplete outcome data, (6) selective reporting, and (7) other potential sources of bias. Each domain was classified as presenting a low, high, or unclear risk of bias based on the information reported in the respective articles. The assessments were performed independently by two reviewers, with any disagreements resolved through discussion or consultation with a third reviewer.

## 3 Results

**Figure 1:**
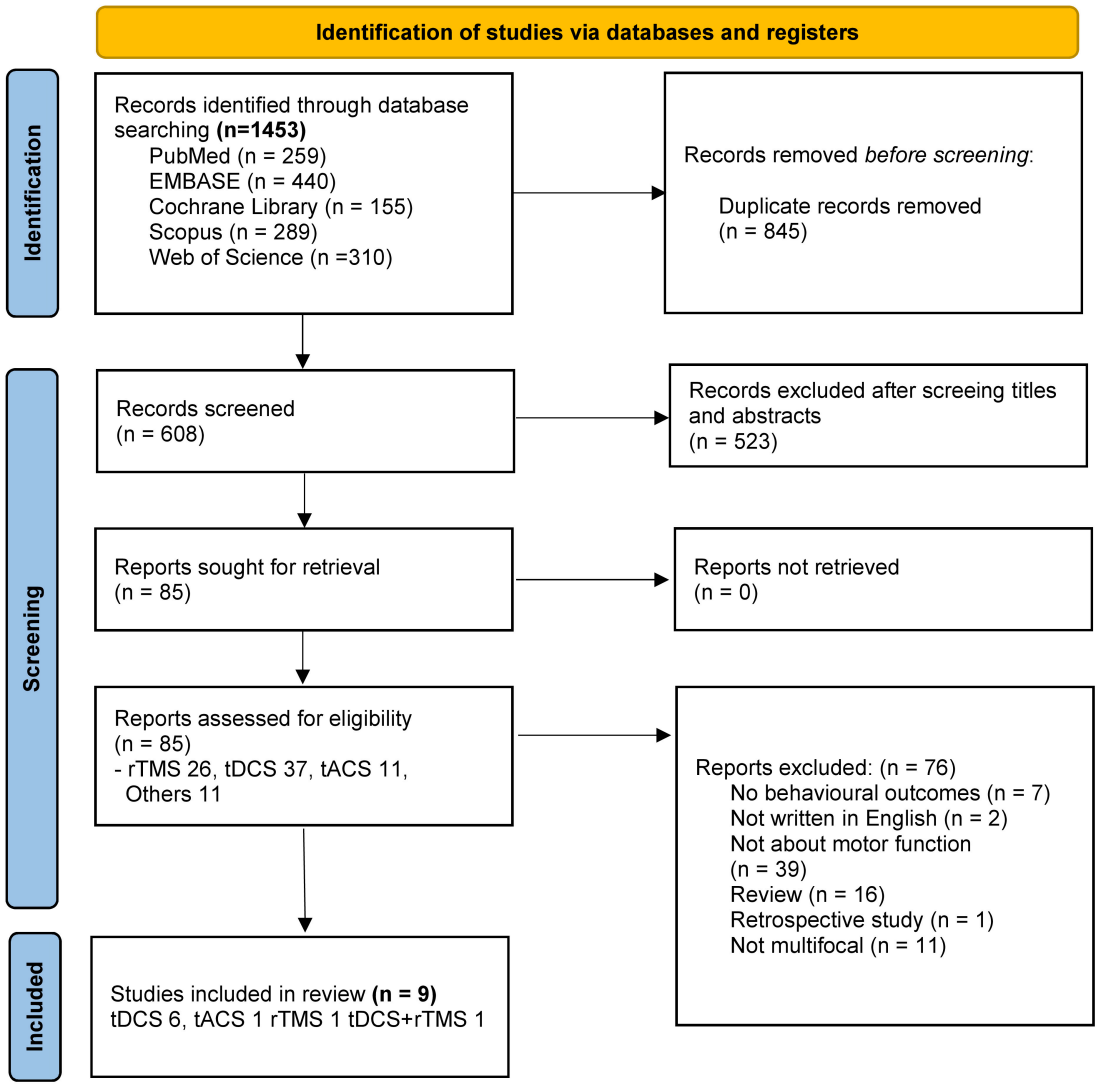
PRISMA flow chart of study selection.

The study selection process is demonstrated in Figure 1. A total of 1,453 records were retrieved through the database searches. After excluding duplicates, 608 records remained, which underwent screening by their titles and abstracts. A total of 85 full-text articles were assessed with full text. Among these articles, 76 were excluded for the various reasons as documented in the flowchart, resulting in 9 studies utilizing t DCS (*n* = 6), tACS (*n* = 1), rTMS (*n* = 1) and a comparison of rTMS and tDCS dual stimulation and rTMS mono-stimulation (*n* = 1) included in the review in final.

**Figure 2:**
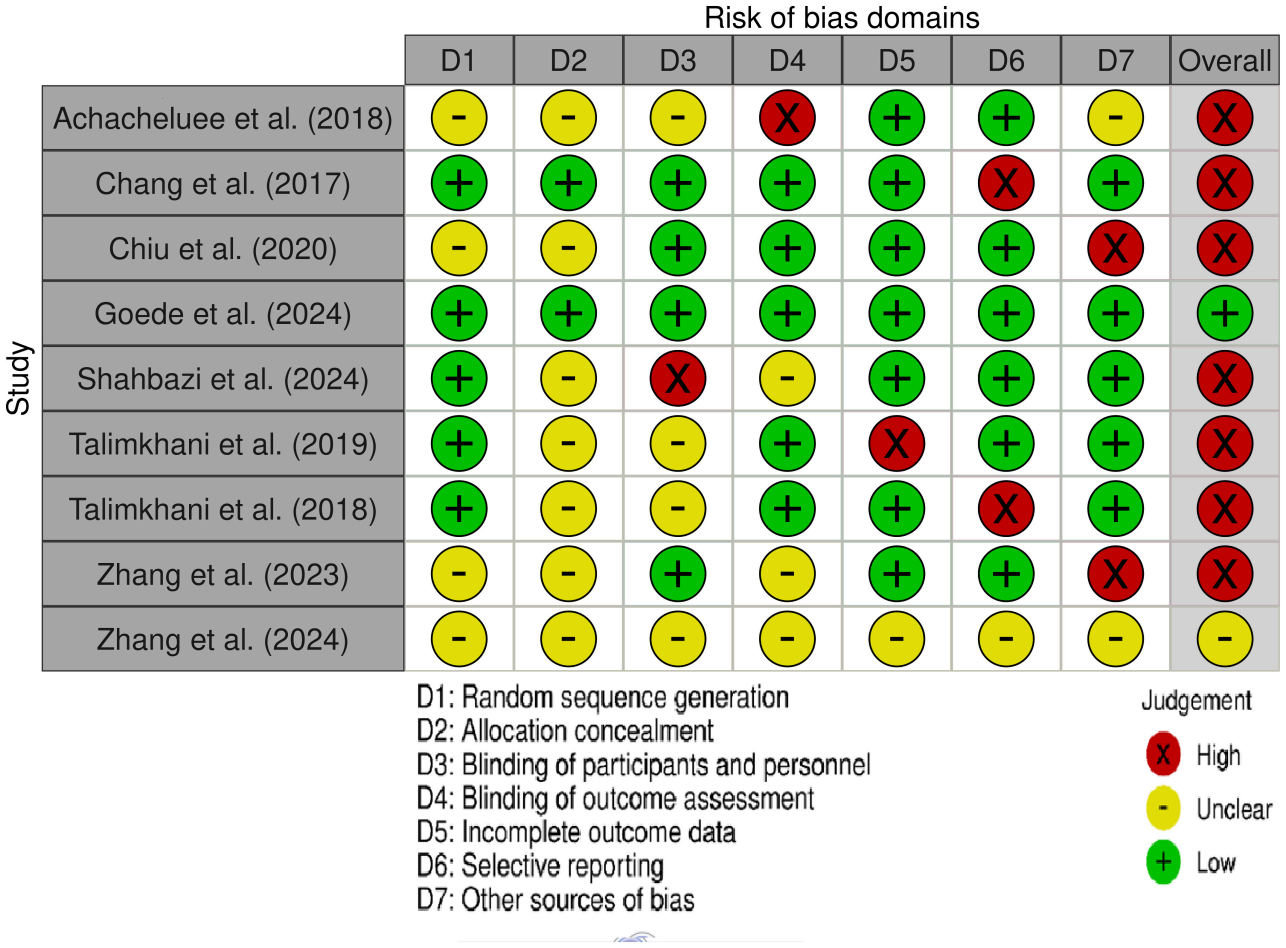
Risk of Bias assessment of the studies included.

Risk of bias was found to differ among the included studies (Figure 2). While most studies exhibited a low risk in domains concerning random sequence generation and incomplete outcome data, the methods for allocation concealment and blinding were often insufficiently reported, leading to an unclear assessment in these areas. In addition, selective outcome reporting frequently remained unclear chiefly due to the lack of access to pre-registered protocols. Ultimately, only one study was deemed to have low risk of bias in every domain, whereas all other studies were judged to have at least one domain rated as unclear or high risk

### 3.1 Multifocal non-invasive brain stimulation targeting motor function

Given the high heterogeneity of the included studies—encompassing diverse stimulation modalities, target combinations, participant populations, and outcome measures—quantitative meta-analysis was not feasible, and a descriptive synthesis was performed. To facilitate interpretation, the nine included studies can be broadly categorized by their stimulation target combination: (1) DLPFC and M1 co-stimulation in healthy participants or those with early neurological impairment (Achacheluee et al., 2018; Talimkhani et al., 2018, 2019; Shahbazi et al., 2024); (2) M1 and cerebellar co-stimulation in high-performing athletes (Zhang et al., 2024); (3) bilateral or multifocal cortical motor network stimulation in persons with stroke (Chiu et al., 2020); (4) dual-modality stimulation targeting DLPFC and M1 in persons with Parkinson's disease experiencing freezing of gait (Chang et al., 2017); (5) network-based multifocal tDCS guided by invasive neuromodulation mapping in persons with Parkinson's disease (Goede et al., 2024); and (6) frontoparietal tACS targeting motor imagery in a brain-computer interface context (Zhang et al., 2023). Across these categories, the stimulation effects were generally modest and population-specific, with some studies demonstrating clear advantages of multifocal over monofocal stimulation and others reporting comparable or inferior effects. The individual study findings are presented in detail below.

The nine studies targeting motor function included six using tDCS (Achacheluee et al., 2018; Talimkhani et al., 2018, 2019; Goede et al., 2024; Shahbazi et al., 2024; Zhang et al., 2024), one using tACS (Zhang et al., 2023), one using rTMS (Chiu et al., 2020), and one combining rTMS and tDCS (Chang et al., 2017) (Tables 1, 2). Two studies by Talimkhani investigated the effects of online tDCS on motor learning in healthy participants over three daily sessions using a parallel design. Both targeted the left DLPFC and M1 with 1 mA of anodal tDCS, while the participants performed a serial response time task. The task involved responding with all four fingers to visual stimuli presented in a sequential pattern (12 sequences across four positions) repeated in 10 training blocks. The skill index, calculated as the ratio of correct responses to the mean response time, was used as the primary outcome measure. Both studies found no significant differences between the stimulation and sham groups immediately after the three-day training period. However, the retention of motor learning was significantly better in the dual stimulation group at follow-up assessments, with (Talimkhani et al. 2018) showing a significantly higher skill index at 1 week (*d* = 0.62; SI: 0.36 ± 0.03 vs. 0.29 ± 0.02 [mean ± SEM]) (Talimkhani et al., 2018) and (Talimkhani et al. 2019) at 1 month (d = 0.67; SI: 0.36 ± 0.03 vs. 0.29 ± 0.02 [mean ± SEM]) (Talimkhani et al., 2019). Notably, data from (Talimkhani et al. 2019) indicated that, while the dual stimulation group exhibited higher error rates than the sham group, their faster response times outweighed the errors, resulting in a better skill index (Talimkhani et al., 2019). None of the studies used neurophysiological measurements.

**Table 1:** Multifocal non-invasive brain stimulation studies targeting motor function.

Study	Intervention	Stimulation montage	Intensity	Frequency	Duration	Target function	Participants	Design	Session
(Achacheluee et al. 2018)	Offline tDCS	M1: (C3—Fp2) or (C4—Fp1); DLPFC: (F3—Fp2) or (F4—Fp1) (active at ipsilesional)	1 mA	N/A	20 min	Motor recovery	15 stroke patients (6F, age 65.03 ± 6.43)	Crossover—dual-site—M1—sham	1 (1wk washout)
(Chang et al. 2017)	Offline rTMS & tDCS	M1: hotspot for Rt TA; l-DLPFC: F3—Fp2	90% RMT (rTMS); 1 mA (tDCS)	10 Hz (rTMS)	20 min	Motor (FOG); Cognition	32 PD patients with FOG (12F, age 63.7 ± 7.9)	Parallel—dual-site: 16—M1 rTMS: 16	5/5 days
(Chiu et al. 2020)	Offline rTMS	CL-M1: 1cm ānd 4cm lateral to midline; IL-M1: 2 points around the lesion; IL-LPC: 5cm anterior to C3 or C4; IL-SMA: 15% nasion-inion anterior to Cz;	< RMT	5 Hz (IL) 0.2 Hz (CL)	40 min	Motor recovery	29 stroke patients (15F, age 65.83 ± 10.98)	Parallel—TRPMS: 14—sham: 15	20/4 wks
(Goede et al. 2024)	Offline tDCS	- anode: (C1, C2, C3, C5, FC6, PO3, PO4, Cz)—cathod: (CP3, CP4, FC1, FC2, FP2, Oz); derived from prior STN-DBS study	< 4 mA (total)	N/A	20 min	Motor symptom	21 PD patients (4F, age 59.7 ± 6.1)	Crossover—active—sham	1 (~24 h washout)
(Shahbazi et al. 2024)	Offline tDCS	bilateral M1: Cz—AF4 l-DLPFC: F3—(between Fpz ānd AFz)	2 mA	N/A	20 min	Motor coordination	36 sedentary adolescent girls (age 16.00 ± 1.05)	Parallel—a-tDCS+VR: 12—sham+VR: 12—control: 12	12/4 wks
(Talimkhani et al. 2019)	Online tDCS	l-DLPFC: F3—Fp2; l-M1: C3—Fp2	1 mA	N/A	20 min x 8 blocks	Motor learning	53 healthy adults (40F, age 27.94 ± 3.81)	Parallel—dual-site: 20—M1: 16—sham: 17	3/3 days
(Talimkhani et al. 2018)	Online tDCS	l-DLPFC: F3—Fp2; l-M1: C3—Fp2	1 mA	N/A	20 min x 8 blocks	Motor learning	37 healthy adults (27F, age 27.87 ± 3.68)	Parallel—dual-site: 20—sham: 17	3/3 days
(Zhang et al. 2023)	Offline tACS	r-PFC: F4—Cz; r-Parietal: P4—Cz	1 mA	10 Hz (α)	20 min	Motor imagery (MI)	36 healthy adults (14F, age 24.6 ± 2.1)	Parallel—in-phase: 12—anti-phase: 12—sham: 12	1
(Zhang et al. 2024)	Offline tDCS	M1: Cz—above the nasion CB: 1cm inferior to the inion—left deltoid	2 mA	N/A	15 min	Motor performance (shooting accuracy, static & dynamic balance)	21 elite ice hockey players (8F, age 21 ± 2.52)	Crossover—dual-site—M1—CB—sham	1 (72h washout)

CB, cerebellum; CL, contralesional; DLPFC, dorsolateral prefrontal cortex; F, females; FOG, freezing of gait; IL, ipsilesional; LPC, lateral premotor cortex; M1, primary motor cortex; PD, Parkinson's disease; PFC, prefrontal cortex; RMT, resting motor theshold; rTMS, repetitive transcranial magnetic stimulation; SMA, supplementary motor area; STN-DBS, subthalamic nucleus deep brain stimulation; TA, Tibialis anterior; tACS, transcranial alternating current stimulation; tDCS, transcranial direct current stimulation; TPRMS, transcranial rotating permanent magnet stimulator stimulation; VR, virtual reality.

**Table 2:** Results of multifocal noninvasive brain stimulation studies targeting motor function.

Study	Main outcome measurement	Measurement timing	Behavioral result	Neurophysiological result
(Achacheluee et al. 2018)	RT to visual stimuli, 9-PPB, FMA-UE	POST (immediate)	RT↓ (vs. sham) (*d* = 0.53), 9-PPB time↓ (*d* = −0.21) (vs. M1 & sham tDCS) by DLPFC-M1 tDCS (RT: 0.577 ± 0.037 vs. 0.665 ± 0.048 s; 9-PPB: 65.75 ± 5.47 vs. 70.38 ± 5.96 s)	N/A
(Chang et al. 2017)	FOG-Q, modified Standing-start 10′ Turn Test, UPDRS-III, TUG; DST (forward & backward), TMT-B, K-MoCA, GDS-SF, MEP	POST (immediate); POST (1 wk)	No inter-group difference in motor function. TMT-B↓ (*d* = 0.44)by DLPFC-M1 stimulation (50.5 ± 21.8 to 41.6 ± 15.2 vs. 49.7 ± 18.4 to 49.0 ± 18.3 s) (vs. M1 rTMS)	No inter-group difference
(Chiu et al. 2020)	FMA-UE, ARAT, grip & pinch strength, TUG, NIHSS; task-related fMRI (M1 during gripping)	POST (immediate); POST (1 wk); POST (1 mo)	No inter-group difference	ΔNumber of active voxels↑(immediate, 1 mo), ΔIntensity of active voxels↑ (immediate);
(Goede et al. 2024)	UPDRS-III, rotating hand movement task (velocity, frequency)	POST (immediate), POST (1 h)	UPDRS-III ↓ (η^2^ = 0.053) active tDCS (vs. sham); Δ = −3.62 ± 5.29 vs. −0.52 ± 6.11 * Post-trial DBS effect ∞tDCS effect	N/A
(Shahbazi et al. 2024)	EHC (100-error/time), BC (100-error/time)	POST (24 h), POST (2 wk)	EHC↑ (d = 1.04) at 2 wk by a-tDCS+VR (vs sham+VR) (1.98 ± 0.39 vs. 1.51 ± 0.48) BC↑(d = 2.3) at 2 wk by a-tDCS+VR (vs sham+VR) (2.39 ± 0.42 vs. 1.50 ± 0.36) EHC↑, BC↑ at 24 h by a-tDCS & sham-tDCS (vs. control) ΔBC (2 wk-−24 h) (d = 0.9) ↑ by a-tDCS+VR	N/A
(Talimkhani et al. 2019)	serial response time task (SI)	POST (immediate); POST (1 mo)	SI↑ (d = 0.67) at 1 mo by DLPFC-M1 tDCS (0.36 ± 0.03 vs. 0.29 ± 0.02) (vs. sham)	N/A
(Talimkhani et al. 2018)	serial response time task (SI)	POST (immediate); POST (1 wk)	SI↑ (d = 0.62) at 1 wk by DLPFC-M1 tDCS (0.36 ± 0.03 vs. 0.29 ± 0.02) (vs. sham)	N/A
(Zhang et al. 2023)	MI-BCI performance (CA) for simple & complex task; task-related EEG (μ-ERD, FC by wPPC in frontoparietal network)	POST (immediate)	CA↑ (d = 0.81) for complex task by anti-phase tACS (88.31 ± 7.04% vs. 79.71 ± 13.17%)	μ-ERD↑ during complex task by anti-phase tACS; Frontoparietal FC in μ ↓ during complex task by anti-phase tACS.
(Zhang et al. 2024)	Ice hocking shooting on unstable flatform (SA), stance test (2-legged stance with eyes open & closed, 1-legged stance with eyes open; EA), proprioceptive assessment (TE), Y-Balance test (composite score)	POST (immediate)	EA ↓ by dual-site, M1, CB a-tDCS for 2-legged eye-closed stance & dominant 1-legged stance (vs. sham) EA ↓ by dual-site, M1 for nondominant 1-legged stance (vs. sham) TE ↓ by dual-site for both legs, and M1 a-tDCS for dominant leg (vs. sham) Composite score ↑ by dual-site, M1 (vs. sham), CB a-tDCS (vs.sham, vs. dual-site) for dominant leg SA ↑ by M1, CB a-tDCS (vs. sham)	N/A

9-PPB, nine-pin pegboard; ARAT, Action Research Arm Test; BC, bimanual coordination; BCI, brain-computer interface; CA, classification accuracy; DBS, deep brain stimulation; DLPFC, dorsolateral prefrontal cortex; DST, digit span test; EA, ellipse area; EEG, electroencephalography; EHC, eye-hand coordination; ER, error rate; ERD, event-related desynchronization; FC, functional connectivity; FMA-UE, Fugl-Meyer assessment of upper extremity; FOG-Q, freezing of gait quiestionnaire; GDS-SF, Geriatric Depression Scale Short Form; K-MoCA, Korean version of Montreal Cognitive Assessment; M1, primary motor cortex; MEP, motor evoked potential; NIHSS, National Institutes of Health Stroke Scale; POST, postintervention; RT, reaction time; rTMS, repetitive transcranial magnetic stimulation; SA, shooting accuracy; SI, Skill Index = (% of correct sequence/mean response time); tACS, transcranial alternating current stimulation; tDCS, transcranial direct current stimulation; TE, trace error; TMT-B, trail making test part B; TUG, timed up and go test; UPDRS-III, Unified Parkinson's Disease Rating Scale part III; VR, virtual reality; wPPC, weighted pairwise phase consistency.

The asterisk (*) indicates an additional note.

(Shahbazi et al. 2024) investigated the effects of dual-site anodal tDCS targeting the left DLPFC and bilateral M1, combined with virtual reality (VR) exergaming on motor coordination in sedentary adolescent girls (Shahbazi et al., 2024). Thirty-six participants were randomly assigned to the active tDCS + VR, sham tDCS + VR, or control group. The active stimulation protocol involved 2 mA anodal current over F3 and Cz, with cathodes at AF4 and Fpz–AFz applied for 20 min prior to 60 min of VR-based gameplay using Xbox Kinect. The sessions were conducted 3 times weekly for 4 weeks. Eye–hand coordination (EHC) and bimanual coordination (BC) were assessed at baseline, 24 h post-intervention, and 2 weeks later using standardized tracing tasks. Two-way mixed ANOVA revealed significant group × time interactions for both outcomes. Although both the active and sham tDCS groups (who received VR training) showed improvements relative to the control group, the active tDCS group demonstrated significantly greater retention effects than the sham group (EHC: *d* = 1.04; 1.98 ± 0.39 vs. 1.51 ± 0.48; BC: *d* = 2.3; 2.39 ± 0.42 vs. 1.50 ± 0.36 [mean ± SD]), and BC continued to improve during the retention period (*d* = 0.9). To date, no neurophysiological outcomes have been reported.

(Zhang et al. 2024) conducted a randomized crossover study in 21 elite ice hockey players to simultaneously examine the effects of anodal tDCS over the M1, the cerebellum (CB), and both sites on balance and shooting accuracy (Zhang et al., 2024). Following each stimulation condition (M1, CB, dual-site, sham), the participants completed a battery of motor tasks: static balance (elliptical area during 2-legged and 1-legged stance), proprioceptive tracing (average trace error), Y-balance test (composite reach score), and shooting accuracy task. The results showed that both monofocal and dual-site stimulations improved various components of balance, with task-specific differences. For example, all active conditions significantly improved 2-legged stance balance with eyes open (*d* = 0.75–1.06), and 1-legged stance with the dominant leg (*d* = 0.64–0.79), but only dual-site and M1 stimulation were effective for the non-dominant leg. In the dynamic balance tasks, trace errors and Y-balance scores improved under the M1 and dual-site conditions, although cerebellar stimulation showed the greatest effect on Y-balance (*d* = 1.28). In contrast, shooting accuracy improved only under M1 and CB stimulation (*d* = 0.81, 0.92) but not under dual-site stimulation, suggesting non-additive or task-interfering effects.

Two studies investigated the effects of motor recovery interventions in persons with stroke (Achacheluee et al., 2018; Chiu et al., 2020). (Achacheluee et al. 2018) conducted a crossover study in individuals with subacute subcortical ischemic stroke, comparing dual-site anodal tDCS (1 mA) targeting the ipsilesional DLPFC and M1 for 20 min with sham and single-site M1 stimulation (Achacheluee et al., 2018). The outcomes included the reaction time to visual stimuli, the 9-pin pegboard task completion time, and Fugl-Meyer Assessment (FMA) score changes. The results revealed that reaction time was was shorter with dual stimulation than with sham stimulation (*d* = 0.53; RT: 0.577 ± 0.037 s vs. 0.665 ± 0.048 s [mean ± SE]), and pegboard task completion time was shorter with dual stimulation than with both sham (*d* = −0.21; 9-PPB: 65.75 ± 5.47 s vs. 70.38 ± 5.96 s [mean ± SE]) and single-site stimulations. However, no significant differences were observed in FMA scores between the groups. (Chiu et al. 2020) employed a transcranial rotating permanent magnet stimulator (TRPMS) in persons with chronic ischemic stroke to stimulate the bilateral M1, ipsilesional lateral premotor cortex, and supplementary motor area daily for 40 min over 20 day (Chiu et al., 2020). Excitatory stimulation at 5 Hz was applied to the ipsilesional side, whereas inhibitory stimulation (0.2 Hz) was applied to the contralesional M1. The primary outcomes were changes in the active voxel count and intensity during gripping tasks, measured using fMRI. Compared to the sham group, the active stimulation group demonstrated a significantly greater increase in the active voxel count at 1 day post-intervention (median change: + 48.5 vs. −30) and at 30 days post-intervention (median change: + 5.5 vs. −87.5). The post-treatment total active voxel count was also significantly higher in the active stimulation group at 1day post-intervention (median: 227.5 vs. 26). Additionally, the increase in the mean voxel intensity was significantly greater in the active stimulation group at 1 day post-intervention (median *T*-value change: +0.21 vs. −0.33). However, no significant differences were observed between the groups regarding clinical outcomes, including FMA, Action Research Arm Test, grip and pinch strength, Timed Up and Go, and National Institutes of Health Stroke Scale scores.

Two studies investigated the impact of multifocal stimulation on motor symptoms in persons with Parkinson's disease (PD) (Chang et al., 2017; Goede et al., 2024). (Chang et al. 2017) explored the combined effects of tDCS and rTMS on freezing of gait (FOG) in persons with PD (Chang et al., 2017). The study administered 10 Hz rTMS to the tibialis anterior hotspot in the M1 and 1 mA anodal tDCS to the ipsilateral DLPFC simultaneously for 20-min sessions, over five consecutive days. Outcome measures included the FOG questionnaire and the modified standing-start 180° turn test to assess FOG severity, as well as the Timed Up and Go test and Unified Parkinson's Disease Rating Scale Part III (UPDRS-III) to assess general motor and ambulatory functions. Cognitive functions were also evaluated, including attention (digit span: forward), working memory (digit span: backward), executive function (Trail Making Test Part B), global cognitive function (Montreal Cognitive Assessment), and depressive mood (Geriatric Depression Scale-Short Form). Assessments were conducted at baseline, immediately after the intervention, and 1 week post-intervention to compare dual stimulation with rTMS alone. The results revealed significant improvements in motor-related outcomes over time in both groups. However, no significant group differences were observed. Among the cognitive function tests, only the Trail Making Test-Part B demonstrated a group-by-time interaction (d = 0.44; dual-site: 50.5 ± 21.8 s to 41.6 ± 15.2 s vs. rTMS: 49.7 ± 18.4 s to 49.0 ± 18.3 s [mean ± SD]). *Post-hoc* analyses indicated that dual stimulation led to greater improvements immediately after the intervention and at 1 week post-intervention; however, these findings did not survive Bonferroni correction. Regarding cortical excitability, no changes were observed in the motor-evoked potential (MEP) resting motor threshold; however, the MEP amplitude increased significantly over time in both groups; however, no significant between-group difference was observed.

(Goede et al. 2024) developed a multifocal tDCS protocol based on a brain network previously identified by DBS network mapping in persons with PD (Goede et al., 2024). Their previous research (Horn et al., 2017) had delineated a “PD response network” by correlating the volume of tissue activated during subthalamic nucleus (STN)-DBS with clinical improvements. This network included positive connectivity to the SMA, anterior cingulate cortex, and medial prefrontal cortex, and negative connectivity to M1. Utilizing electric field modeling, the authors configured a multifocal tDCS montage that best replicated the connectivity profile. In a double-blind, randomized, crossover trial, 21 persons with PD received 20 min of active or sham tDCS targeting this network on separate days with at least a 24-h washout period. Motor symptoms were assessed using the Movement Disorder Society (MDS)-UPDRS-III. Active stimulation reduced MDS-UPDRS-III scores compared to sham (Δ = −3.62 ± 5.29 vs. −0.52 ± 6.11 [mean ± SD]; η^2^ = 0.053). Notably, motor improvement remained significant 60 min after stimulation only in the active condition. In an exploratory analysis of 11 individuals who subsequently underwent STN-DBS, improvement in tDCS was correlated with postoperative DBS outcomes (*r* = 0.55), suggesting the potential role of multifocal tDCS as a predictive tool for DBS candidacy. No adverse events were reported.

(Zhang et al. 2023) was the sole study to apply tACS, not focusing on direct motor function changes, but on the impact of stimulation on motor imagery performance within a brain-computer interface (MI-BCI) (Zhang et al., 2023). The study administered 1 mA of 10 Hz tACS to the right prefrontal and parietal cortices for 20 min, followed by motor imagery tasks involving the left hand. These included a simple task (gripping) and a complex task (writing). The primary outcome was the change in BCI classification accuracy pre- and post-stimulation compared across in-phase, anti-phase, and sham conditions. For the simple task, no significant differences were observed between the conditions. However, for the complex task, an interaction effect was observed between groups, with *post-hoc* analysis indicating improved classification accuracy exclusively in the anti-phase condition (*d* = 0.81; 88.31% ± 7.04% vs. 79.71% ± 13.17% [mean ± SD]). To further explore the effects of stimulation, the relative changes in the power spectra of the mu and beta frequency bands at the C4 electrode (i.e., averaged event-related desynchronization [ERD] values) were measured. Significant interaction effects were observed only in the mu band during the complex task, with *post-hoc* analysis indicating differences in the anti-phase condition. Additionally, functional connectivity between the right frontal and parietal electrodes was evaluated using weighted pairwise phase consistency (wPPC). Unk-related functional connectivity networks were constructed using normalized wPPC (wPPCz) values derived from the 30 frontoparietal electrodes. Although no interaction effects were identified for the mean wPPC values across conditions, functional connectivity in the bilateral frontoparietal network during the complex task significantly decreased under anti-phase tACS. Notably, the wPPCz value of the contralateral frontoparietal network exhibited a significant difference in the *post-hoc* analysis (*p* < 0.01).

Taken together, the findings across the nine included studies do not support a uniform conclusion regarding the superiority of multifocal NIBS over monofocal approaches for motor outcomes. Studies employing concurrent DLPFC-M1 stimulation consistently demonstrated enhanced long-term retention of motor learning, whereas studies targeting clinical populations—including those with stroke or Parkinson's disease—yielded more variable findings, with several reporting no significant between-group differences in primary motor outcomes. Notably, in one crossover study, dual-site stimulation was even inferior to monosite stimulation for certain motor tasks, highlighting the possibility of non-additive or interfering effects. These heterogeneous findings underscore the importance of considering task type, clinical population, stimulation target combination, and methodological design when evaluating the efficacy of multifocal NIBS. A comprehensive summary of study characteristics and outcomes is provided in Tables 1, 2.

## 4 Discussion

### 4.1 Multifocal non-invasive stimulation targeting motor function

Studies targeting motor function have frequently employed concurrent stimulation of the DLPFC and M1, showing significant effects, primarily in terms of motor task performance speed (Achacheluee et al., 2018; Talimkhani et al., 2018, 2019; Shahbazi et al., 2024), particularly in paradigms involving motor sequence learning and coordination. Notably, the observed differences between the stimulation and sham groups were more pronounced than in studies focusing solely on M1 tDCS, suggesting that additional stimulation of the DLPFC contributed to these effects. The improved long-term retention of motor learning observed in three studies (Talimkhani et al., 2018, 2019; Shahbazi et al., 2024) points to the potential influence of neuromodulation on synaptic plasticity (Vogeti et al., 2022). However, the lack of neurophysiological measurements in these studies limits the strength of our conclusions. Moreover, since these studies did not compare dual-site stimulation with stimulation of the DLPFC alone, it remains unclear whether the effects were due to network-level modulation or simple activation of the DLPFC. Previous studies reported that DLPFC activity can influence motor performance and cognitive speed (Gbadeyan et al., 2016; Lee et al., 2016), which may explain the observed effects. Further studies that incorporate neurophysiological measurements and direct comparisons with DLPFC-only stimulation are required to elucidate the mechanisms underlying these findings.

Notably, none of the studies employing DLPFC-M1 multifocal stimulation included neurophysiological measurements of corticospinal excitability, such as motor evoked potentials (MEPs). MEP amplitude and resting motor threshold (RMT), obtained through single-pulse TMS, are established indices of corticospinal excitability and have been widely used to assess the neurophysiological effects of tDCS in prior work. Their systematic inclusion in future multifocal NIBS studies targeting motor learning would be highly valuable. Specifically, comparing MEP changes between DLPFC-M1 dual-site and M1-only stimulation conditions would allow researchers to determine whether dual-site stimulation enhances corticospinal excitability beyond what M1 stimulation alone achieves, thereby providing mechanistic evidence for network-level effects on the motor system. Furthermore, assessing the relationship between stimulation-induced MEP changes and behavioral improvements in motor learning and retention would help clarify whether corticospinal excitability modulation underlies the observed long-term retention benefits.

(Zhang et al. 2024) used a fully crossed, within-subject design to compare dual-site anodal tDCS over M1 and the cerebellum to both monosite stimulation and sham stimulation in a high-performing athlete sample (Zhang et al., 2024). The effects of stimulation were generally more pronounced in the dominant leg, and no significant improvements were observed in the two-legged stance with eyes closed or the Y-balance test using the non-dominant leg. While dual-site stimulation was superior in certain tasks, such as one-legged stance and proprioceptive tracing with the non-dominant leg, it was unexpectedly less effective than monosite stimulation for shooting accuracy on an unstable platform. Most observed effect sizes were in the moderate range (Cohen's *d* = 0.6–1.2). These task-dependent patterns likely reflect differences in balance control strategy, sway amplitude, and motor complexity; however, interpretation is limited by the absence of neurophysiological data. Moreover, although performance was normalized to baseline, the use of repeated outcome tasks across sessions and a fixed stimulation order (with the initial condition determined by the enrollment sequence) meant that true randomization and allocation concealment were not ensured and potential order effects were not assessed, posing a methodological limitation.

The study by (Goede et al. 2024) is noteworthy in that it applied a multifocal tDCS montage designed to replicate the connectivity profile of a “Parkinson's disease response network” previously identified through DBS-based mapping (Horn et al., 2017). This approach demonstrated that non-invasive stimulation of surface regions connected to effective DBS targets could produce clinical improvements in UPDRS-III scores, and that these improvements correlated with participants' subsequent responsiveness to DBS. This method parallels the network-based montage construction used by (Vaqué-Alcázar et al. 2021) in the cognitive domain and offers a promising strategy for targeting complex functional systems using individualized structural or functional MRI-derived connectivity maps. Moreover, these findings suggest that multifocal tDCS may indirectly modulate deep brain structures via associated networks, underscoring its potential translational value. However, the absence of neurophysiological measurements and relatively large contribution of a subset of responders to the observed effects highlight the need for further research to identify the optimal patient profile for such interventions.

Two studies incorporated rTMS into their design (Chang et al., 2017; Chiu et al., 2020). Since we excluded ccPAS and priming stimulation studies, the remaining studies involving rTMS were limited to two: one used a specialized device, TRPMS (Chiu et al., 2020), and one combined rTMS and tDCS (Chang et al., 2017). (Chiu et al. 2020) demonstrated that four weeks of TRPMS expanded the size and activation of motor maps compared to sham, as observed through fMRI (Chiu et al., 2020). However, no statistically significant improvements in behavioral outcomes have been reported. This study primarily served as a feasibility investigation and did not clarify connectivity changes or highlight the unique advantages of multifocal stimulation. (Chang et al. 2017) investigated dual-site stimulation involving tDCS over the DLPFC and rTMS over M1 in individuals with parkinsonism who experienced FOG (Chang et al., 2017). This study compared dual stimulation with M1 rTMS alone (without sham stimulation) and found that both approaches effectively improved motor symptoms; however, no significant differences were observed between the groups. Notably, dual stimulation was more effective in the cognitive domain, specifically in the Trail Making Test Part B. However, it remains unclear whether these effects were due to stimulation of the DLPFC alone or were unique to the dual-site stimulation setup. Further studies are required to delineate the specific contributions of DLPFC involvement and the added value of concurrent stimulation.

Among the nine included studies, MEP measurements were reported in only one study—(Chang et al. 2017)—and this study found no significant between-group difference in MEP amplitude despite a significant within-group increase over time, suggesting that both rTMS and dual-site stimulation similarly elevated corticospinal excitability without differential effects attributable to the added tDCS. The remaining eight studies did not include any MEP or corticospinal excitability measures. This is a notable limitation given that MEPs represent the most direct and validated non-invasive index of corticospinal tract integrity and plasticity. For studies targeting clinical populations such as stroke or Parkinson's disease, in which corticospinal excitability is often pathologically altered, the absence of MEP data makes it difficult to determine whether behavioral improvements reflect genuine neuroplastic changes at the motor cortex or compensatory mechanisms elsewhere. Future multifocal NIBS trials should, where feasible, incorporate MEP amplitude, cortical silent period, and short-interval intracortical inhibition (SICI) measurements as neurophysiological endpoints, alongside behavioral outcomes. Such measures would substantially strengthen the mechanistic interpretation of any observed effects and enhance the reliability of conclusions drawn from systematic reviews of this literature.

The study by (Zhang et al. 2023), which targeted the frontoparietal network to assess MI-BCI classification accuracy (Zhang et al., 2023), is particularly noteworthy. Although no direct head-to-head comparison was made among the in-phase, anti-phase, and sham tACS conditions, a significant main effect of time was exclusively observed in the anti-phase condition. Given that the baseline values showed no differences across the groups, this indirectly supports the efficacy of anti-phase stimulation. Notably, during the complex task, anti-phase tACS demonstrated a reduction in functional connectivity within the mu band, a neurophysiological effect accompanied by improved classification accuracy, distinguishing between resting-state and task-related activities. Additionally, the study showed enhanced ERD, a key metric commonly used in MI-BCI classification (Pfurtscheller and Lopes da Silva, 1999), suggesting that antiphase tACS induces desynchronization within the network, contributing to improved task performance. In contrast, no significant effects were observed during simple tasks such as gripping. This likely reflects the limited involvement of network-level activity in simple motor tasks, indicating that network-based neuromodulation is less effective in these contexts. These findings have important implications for interpreting the results of other studies and guiding future research, particularly for understanding the role of task complexity in the effectiveness of neuromodulation.

It is important to note that not all included studies demonstrated superiority of multifocal over monofocal NIBS. (Chang et al. 2017) found that dual-site stimulation (tDCS over DLPFC combined with rTMS over M1) did not produce significantly greater improvements in motor outcomes compared with rTMS over M1 alone in persons with Parkinson's disease experiencing freezing of gait (Chang et al., 2017). Similarly, (Zhang et al. 2024) observed that dual-site M1-cerebellar tDCS was inferior to monosite stimulation for shooting accuracy in elite ice hockey players (Zhang et al., 2024), suggesting that concurrent stimulation of two targets may not always be synergistic and may, in certain task contexts, produce non-additive or even interfering neural effects. These findings are consistent with emerging evidence suggesting that the benefits of multifocal NIBS depend critically on the functional relationship between targeted regions, the nature of the task, and the degree of network-level integration required. When two stimulated regions do not functionally synergize—or when the task demands predominantly engage a single node—simultaneous co-stimulation may disrupt rather than enhance performance. This interpretation is supported by the observation that multifocal benefits in the included studies were most robust in tasks requiring cross-domain integration, such as motor-cognitive dual tasks (Chang et al., 2017) or motor learning paradigms with long-term retention (Talimkhani et al., 2018, 2019), where prefrontal-motor network engagement is functionally relevant. Future studies employing multi-arm designs that include monosite stimulation conditions for each individual target will be essential to disentangle the independent and synergistic contributions of multifocal stimulation, and to determine for which tasks and populations multifocal NIBS confers a genuine advantage over monofocal approaches.

### 4.2 Limitations

Several limitations of the present review should be acknowledged. The search strategy was primarily designed to capture studies employing the most established multifocal NIBS modalities—tDCS, tACS, and TMS—and may therefore have missed studies utilizing less commonly investigated techniques. In particular, transcranial random noise stimulation (tRNS) and transcranial pulsed ultrasound stimulation (TPS) were not explicitly targeted by the search terms, and studies employing these modalities in a multifocal paradigm for motor function may not have been captured.

## 5 Conclusion and future direction

Research on multifocal NIBS targeting motor functions has predominantly focused on combinations of prefrontal and motor areas, as well as frontoparietal networks. However, many tDCS and rTMS studies have methodological limitations, such as study designs that fail to clearly isolate the effects of multifocal stimulation and a lack of neurophysiological evidence to support their conclusions. To address these issues, future research should adopt multi-arm designs and integrate neurophysiological measurements to better interpret the results. Developing protocols that effectively link changes in neural network activity and connectivity to behavioral outcomes is essential for advancing the field. Additionally, study designs should consider the unique neurophysiological mechanisms of each NIBS modality and include follow-up assessments to evaluate both immediate and long-term effects, as well as offline retention. Emerging NIBS methods, such as temporal interference stimulation and focused ultrasound, have the potential to target deep brain regions multifocally with a favorable safety profile (Pasquinelli et al., 2019; Vassiliadis et al., 2024). These innovations could broaden the range of neural networks that can be modulated, creating new opportunities to explore the effects of NIBS on complex neural systems.
